# A new low-cost, compact, auto-phoropter for refractive assessment in developing countries

**DOI:** 10.1038/s41598-017-14507-5

**Published:** 2017-10-25

**Authors:** Babak Amirsolaimani, Gholam Peyman, Jim Schwiegerling, Arkady Bablumyan, N. Peyghambarian

**Affiliations:** 1 College of Optical Sciences, University of Arizona, Tucson, AZ 85721 USA; 2 TIPD, LLC, 1430 N. 6th Ave, Tucson, AZ 85705 USA

## Abstract

Using a phoropter to measure the refractive error is one of the most commonly used methods by ophthalmologists and optometrists. Here, we demonstrate design and fabrication of a portable automatic phoropter with no need for patient’s feedback. The system is based on three tunable-focus fluidic lenses and thin-film holographic optical elements to perform automatic refractive error measurement and provide a diagnostic prescription without supervision. Three separate lenses are deployed to correct the defocus and astigmatism. The refractive error is measured using a Shack-Hartmann wavefront sensor that calculates the Zernike values of an infrared wavefront emerging from the eye. Holographic optical elements steer the emerging wavefront into the wavefront sensor, while simultaneously providing an unobstructed view for the subject. The power of each lens is controlled by pumping a liquid in and out of the lens chamber using servo motor actuated diaphragm pumps. Spherical and cylindrical correction range of −10 to +10 diopters with 0.1 diopter increments is achieved in less than 15 seconds using wavefront sensor feedback to the pumps. This system can be used in rapid screening of large patient populations especially in the developing countries that lack sufficient facilities and specialist doctors.

## Introduction

Refractive Error (RE) is among the main concerns of public health, with uncorrected RE being the leading cause^1^ of visual impairment. More than 650 million people suffer from insufficient or no refractive correction globally^2^, and about 285 million^3^ of those (8.2 million in US^4^) become visually impaired, resulting in 250 billion dollar productivity loss^5,6^. Monitoring the RE can significantly reduce the risk of vision loss^7^ especially in developing countries, where there is inadequate access to screening facilities^8^. A phoropter is one of the most commonly used instruments by ophthalmologists and optometrists in which trial lenses are the main components to evaluate the refractive error. These lenses are placed one at a time in front of the patient’s eye, while looking at an eye chart at some distance away. This visual acuity examination is time consuming and requires constant and reliable feedback from the patient, who needs to mentally compare the two images that are created by placing different dioptric power lenses in front his/her eyes. This can cause confusion and often leads to inaccuracy of the prescribed power of the glasses. The process is especially difficult for young children, elderly, or mentally handicapped patients who may have reduced attention, poor memories or verbal communication disabilities. Moreover, a large number of lenses are required to cover the most common vision errors such as myopia (nearsightedness), hyperopia (farsightedness), and astigmatism within the required range, making the system difficult to transport. Fluidic lenses are good alternatives to trial lenses for greatly reducing the number of lenses required in a phoropter system. Continuous power change of these lenses is also advantageous for lowering the 0.25-diopter increments of trial lenses. Tunable lenses have been studied in different applications^9–14^ including ophthalmic correction using manually pumped lenses^15–18^. While these hydraulically pumped lenses have shown to support a large correction range, they lack a feedback system for automatic operation in addition to having a large system footprint. Various methods have been investigated to fabricate and control liquid lenses^19–25^ of which externally actuated hydraulic pumps have shown good control and large variations in focal lengths^26,27^. Although most of these methods are limited to spherical powers, recent studies have shown tunable astigmatism in liquid lenses^28,29^ as well. However, they suffer from limited movement range (limited change in focal length) which makes them unsuitable for refractive correction. Employing servo motors and diaphragm pumps in a small 3D printed package provides high tuning accuracy and large focal length variations of the lenses, while reducing the required actuation voltage and system size. At the same time, adaptive optics and wavefront detectors for human eye aberration measurement and correction have been studied^30–34^ for different applications such as autorefractors^35^. While autorefractors are able to provide high resolution RE detection and correction capabilities, they lack the ability of simultaneous data verification through using trial lenses. Hence, the prescribed data of autorefractors should later be fine-tuned by using a phoropter. To overcome this issue, holographic optical elements (HOEs) can be deployed. Due to the compact form factor and the unique diffractive properties of these HOEs, various applications of them have been studied^36^. One main advantage of HOEs is their ability to work in specific wavelength while being transparent to other wavelengths, which enables them to project the pupil onto the wavefront detector without blocking the patient’s view. This would enable subjective verification of the prescribed data using fluidic lenses. Here we report the design and fabrication of a handheld and low-cost auto-phoropter that is based on fluidic lenses. The automatic correction based on the feedback from a wavefront sensor accelerates the RE evaluation. The fluidic lenses are made of Polydimethylsiloxane (PDMS) membranes and are controlled using servo actuated diaphragm pumps. Tunable lenses are deployed for both defocus and astigmatism correction within the range of −10 to +10 diopters. Moreover, we present the design of HOEs so that they redirect the infrared (IR) light while being transparent in visible wavelength in order to keep an unobstructed view for the patient.

## Results

### Design and fabrication of the system

**Figure 1:**
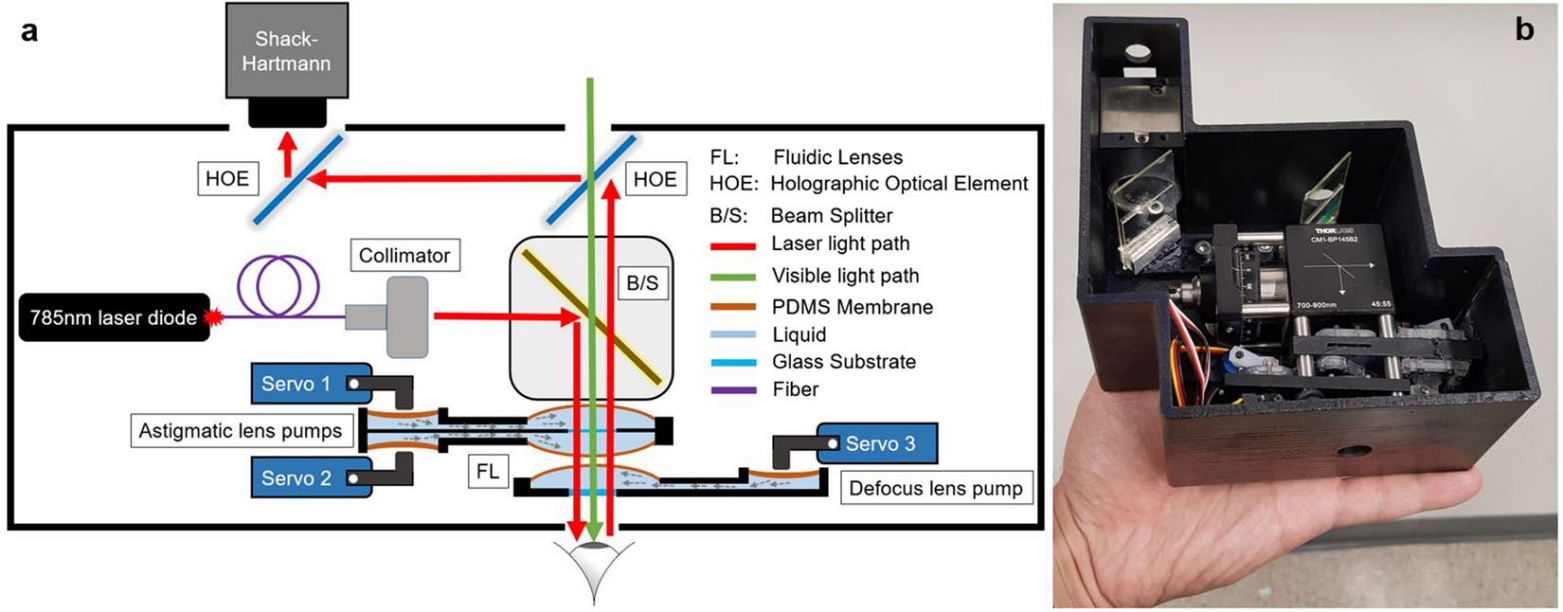
The auto-phoropter setup. **(a)** The red arrow in the schematic diagram shows the optical path of the 785 nm laser light that is used to detect the wavefront aberrations reflecting from retina. Green arrow depicts the patient’s line of sight. The tunable liquid lenses are controlled by servo motor actuated diaphragm pumps to correct the vision errors. **(b)** Fabricated auto-phoropter setup in black enclosure.

The auto-phoropter system schematic is depicted in Fig. 1a. In order to have minimum beam absorption by the water in the eye, the 785 nm IR laser diode is employed as the measurement source. The laser is collimated to create a 7.5 mm diameter beam. The power reaching the eye from the laser is ~0.6 *mW/cm* ^2^, which is less than Maximum Permissible Exposure (MPE) at the cornea^37^ (1 *mW/cm* ^2^). The laser beam is folded 90 degrees using 50:50 beam splitter. The steered beam then passes through the fluidic lenses and reaches the eye. After reflecting back from the retina and going through the tunable lenses, the IR light is redirected by two HOEs into the Shack-Hartmann wavefront sensors. The HOEs also act as an afocal relay to image the eye pupil onto the lenslet array in the wavefront sensor. HOEs are designed specifically for operating at 785 nm while being transparent at visible wavelengths. Hence, patient’s view will not be blocked while looking at a distant eye chart. The distance between the pupil and the first tunable lens is ~15 mm. Figure 1b shows the 3D printed enclosure.

### Fluidic lens setup

**Figure 2:**
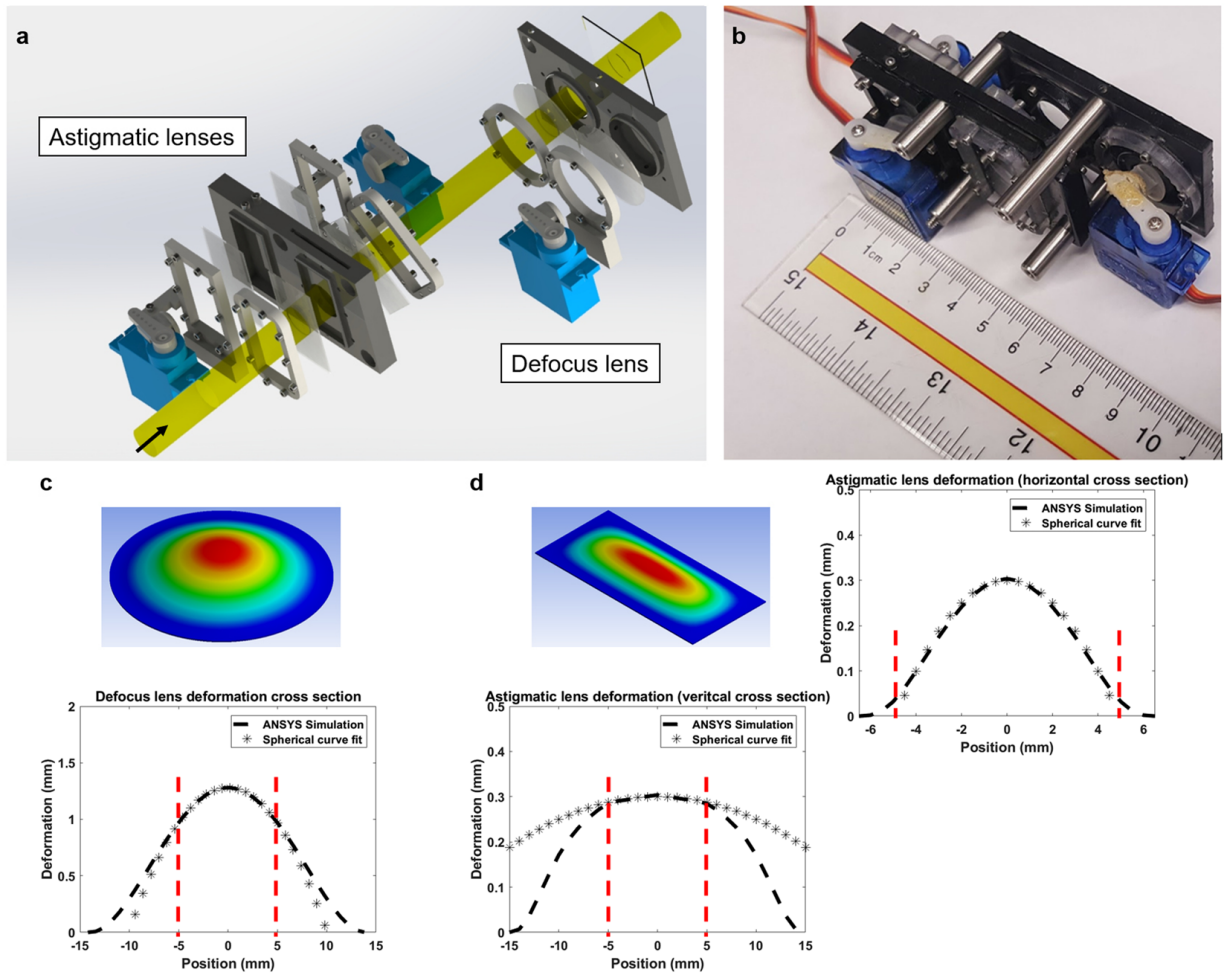
Fluidic lens package. **(a)** Exploded view of the tunable fluidic lenses and pumps. Each lens is controlled using a diaphragm pump that is actuated using a servo motor. The pump diaphragm is made of the same PDMS as the lens membranes. Yellow color illustrates the light path through the lenses. **(b)** The assembled setup including 3D printed structures. **(c)** The simulated membrane deformation for the defocus lens. The fitted spherical curve (40 mm radius) onto the deformation cross section shows that the membrane acts as a spherical surface within the 10 mm diameter optical aperture that is illustrated by the red dashed lines. **(d)** Simulated data for the rectangular membrane fitted with a 40 mm radius curve (10-diopter lens) in the horizontal cross section. Since the radius of curvature in the vertical orientation (1000 mm) is much larger than the horizontal direction, the surface acts as a cylindrical membrane within the optical aperture.

The three separate fluidic lenses that are deployed in the system are shown Fig. 2a. The two cylindrical lenses that are placed at 45 degrees with respect to each other correct the astigmatism, and the third lens (spherical lens) compensates for the defocus. Laser liquid with refractive index of 1.55 is used as the fluid inside the lenses to minimize the index mismatch between the rigid glass side, the fluid, and the deformable PDMS membrane of each lens. Each fluidic lens is tuned individually using a diaphragm pump mechanism that is actuated by a servo motor. Thus, the power increments of each lens are controlled by the step size of the servo motor (~0.1 diopter). The membrane of the pump’s diaphragm is also made of a 600 *μ*m thick PDMS. Encapsulating the lenses and pumps in a 3D-printed package makes the setup compact and reduces the response time of the system. Figure 2b illustrates the fabricated lens setup.

**Figure 3:**
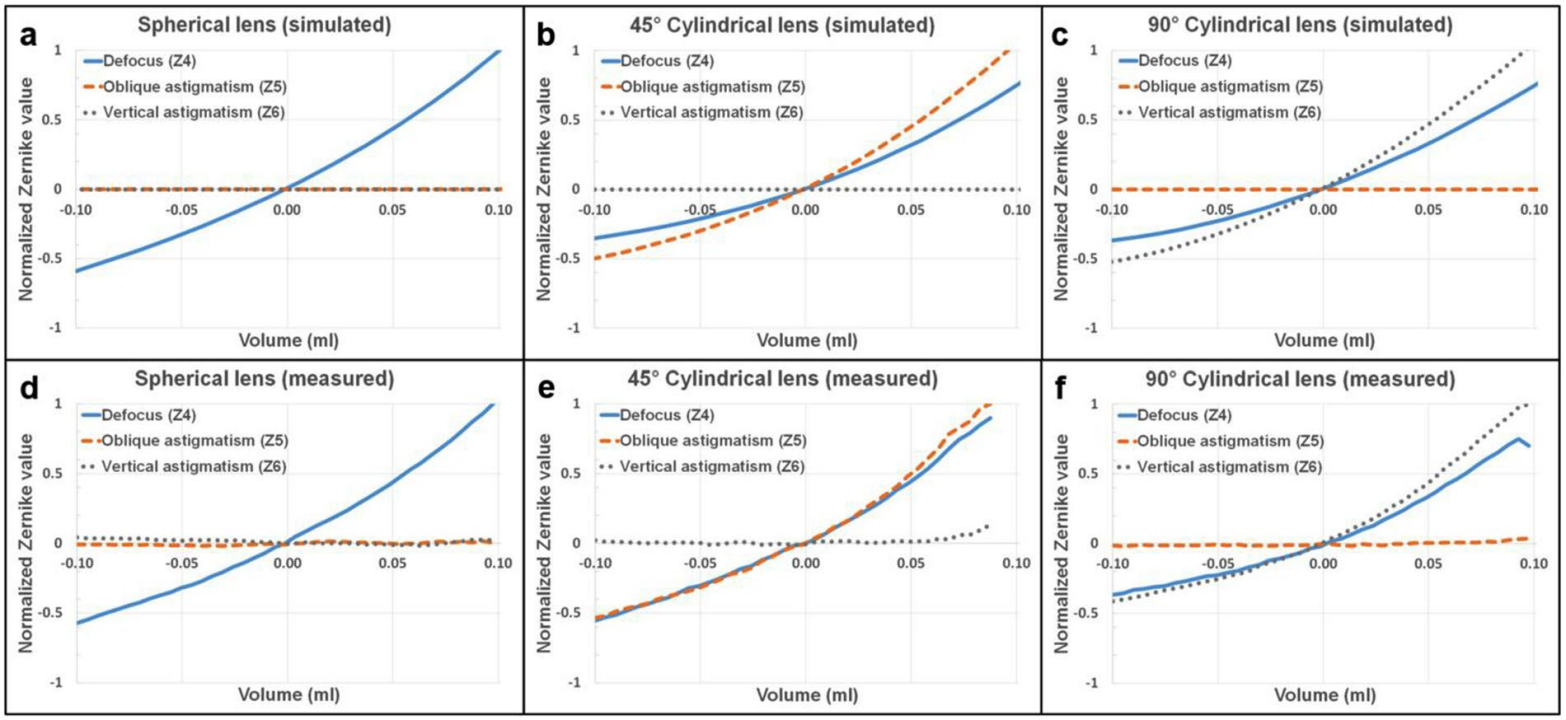
Simulated and measured data of normalized Zernike values. Variations of Zernike values related to defocus (Z4), 45° astigmatism (Z5), and 90° astigmatism (Z6) vs. the volume changes of liquid inside the simulated **(a)** defocus lens, **(b)** 45° astigmatic lens, and **(c)** 90° astigmatic lens. The corresponding measured results are represented for **(d)** defocus lens, **(e)** 45° astigmatic lens, and **(f)** 90° astigmatic lens.

The clear aperture of the lenses is 10 mm in diameter. The deformation of the PDMS membranes can be modeled as a quadratic polynomial^38^. The 3D printed mechanical clamps are chosen such that the lenses behave as spherical or cylindrical within the optical aperture. The PDMS deformation analysis was done to optimize the dimensions of the circular (diameter = 25 mm) and rectangular (width = 13 mm, height = 30 mm) clamps that hold the membranes. Figure 2c depicts the simulated membrane deformation for the defocus lens. The fitted 40 mm spherical surface (10-diopter lens) matches quite well with the simulation data, which shows that the lens performs as a spherical lens within the optical aperture that is illustrated by the red dashed lines. Astigmatic lens dimensions were also chosen such that the lens acts as a cylindrical surface (Fig. 2d). The vertical cross section has much larger radius of curvature (1000 mm) compared to the horizontal cross section (40 mm) within the optical aperture. This corresponds to an error of less than 0.25 diopters compared to an ideal cylindrical lens. To investigate the optical performance, membranes were modeled as spherical and toroidal surfaces for defocus and astigmatism lenses respectively. By changing the pressure on each membrane, the refractive correction can be achieved within the range of −10 to +10 diopters at different angles. Zernike value changes of defocus (Z4), 45° astigmatism (Z5), and 90° astigmatism (Z6) versus the liquid volume change in the spherical lens is represented in Fig. 3a. Figure 3b and c illustrate the same graphs for cylindrical lenses. The volume change measurement is based on the surface Sagitta (SAG) variations of the spherical cap and cylindrical segments (Supplementary note 1). The spherical and cylindrical optometric constants (in diopters) and the axis angle of the cylinder in degrees can be calculated based on these Zernike values (Supplementary note 2).

### HOEs

The pupil is projected on the Shack-Hartmann sensor using a holographic telescope based on two quasi-confocal HOE lenses. The HOEs were fabricated in-house as a volumetric hologram recorded using interferometric pattern of spherical and collimated beams. Volumetric holograms provide high Diffraction Efficiency (DE > 95%) in the specific wavelength range and remain transparent in other wavelengths. Thus, it allows the patient’s view of the test-chart to be clear in the visible spectrum, while the infrared probe beam for the wavefront sensor is efficiently diverted to the Shack-Hartmann sensor. However, holographic materials where such a hologram can be recorded (phase materials) are not sensitive in the IR region. To make distortion-free holographic lenses working at 785 nm wavelength, the same wavelength needs to be used for recording. To fabricate aberration free holographic lenses, an amplitude-type hologram was recorded on IR sensitive photo-emulsion using the geometry of the phoropter setup (Fig. 1a). Next, a contact copy was made from this hologram on a phase material (photo-polymer in our case) using a 532 nm collimated beam. The diffraction pattern in the output of the hologram remains the same, independent of the shape and wavelength of the readout beam. As a result, the hologram made in such a copying process reconstructs a spherical beam similar to the object beam of the master when illuminated with a collimated 785 nm light source. Moreover, due to the similarity of write/read geometries, such reconstruction will satisfy the Bragg condition and thus provides close to 100% DE.

To measure the properties of fluidic lenses, the Zernike value changes are measured separately for each lens using the Shack-Hartmann sensor. As can be seen in Fig. 3d, changing the power of the spherical lens does not affect the Zernike values related to the astigmatism (Z5, Z6). Figure 3e and f represent the changes in Zernike values for the volume changes in oblique and vertical cylindrical lenses respectively. These figures indicate that the measured results match the Zemax model quite well, which shows the fluidic lenses can replace the traditional lens set of a phoropter.

**Figure 4:**
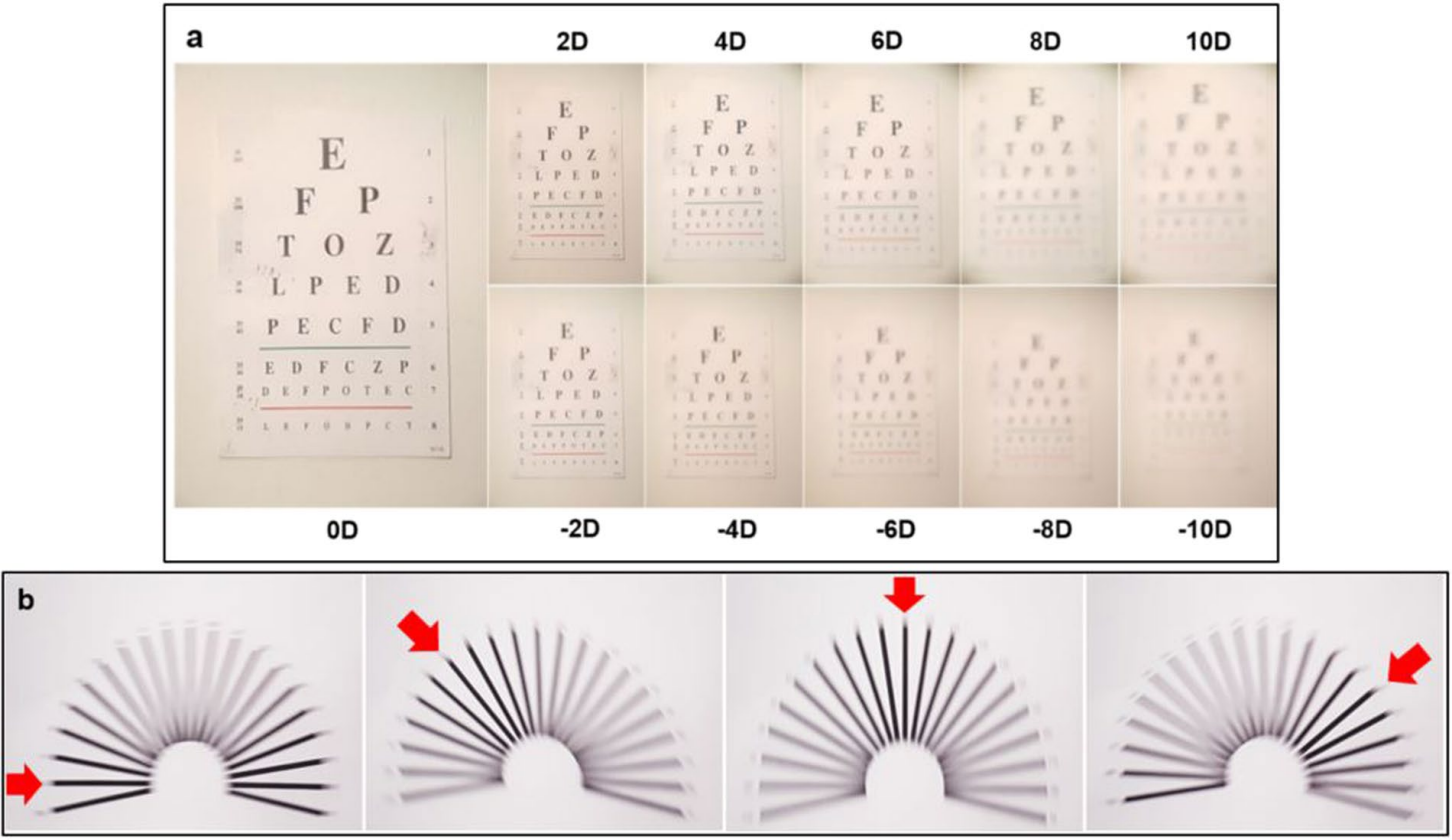
Test chart images captured through the fluidic lens setup. **(a)** The power of the defocus lens is changed from −10 to +10 diopters with two-diopter increments using the servo motor actuated pump. The increments can be as low as 0.1 diopters. **(b)** Astigmatism chart images. Three-diopter magnitude astigmatism is induced at 0°, 50°, 90°, and 140° using cylindrical lenses placed at 45 degrees with respect to each other. The angle can be controlled by changing the ratio between Z5 and Z6.

Figure 4a illustrates eye chart images captured through the fluidic lens setup. Dioptric power variation of −10 to 10 diopters with 2 diopter increments is achieved by changing the liquid volume inside the spherical lens using the servo actuated pump. The step size resolution of the digital servo motor enables the control of the lens power in ~0.1 diopter steps. The cylindrical lenses are also characterized with an astigmatism test chart. Three-diopter induced astigmatism images at different angles are represented in Fig. 4b. The astigmatism angle is controlled by changing the ratio between Z5 and Z6.

To test and calibrate the phoropter setup, the Arizona eye model has been used (Supplementary note 3). The laser diode and the servo motors are controlled through a USB channel. An interactive user interface is also developed to automatically control the pumps and correct the refractive error based on the Zernike values from the Shack-Hartmann sensor. The correction process stops when the refractive error amplitude becomes less than 0.25 diopters (Supplementary Video 1).

## Discussion

The reliable screening of refractive error is one of the challenging goals of the World Health Organization^39^, especially in areas with little or no access to examination facilities. Screening large population of patients such as in schools is another essential issue. In this report, we introduced a compact and low-power auto-phoropter system for rapid evaluation of the RE. Although subjective perception of refractive error can differ from the objective RE evaluation using the Shack-Hartmann detector, these differences are usually limited to ±0.5 diopters^40,41^. To correct and fine-tune the results for these subjective variations, manual control of each lens is also provided in the user interface.

The refractive correction using PDMS-based tunable fluidic lenses showed high accuracy for spherical aberration and astigmatism. 3D printing of the pump and lens chambers on the same structure provide small footprint and increase the accuracy and response time of the system. Although the IR wavefront goes through the fluidic lenses twice, the effect of this double passing can be compensated in the software (Supplementary note 4). Moreover, employing holographic optical elements exploits their unique property in steering the IR light towards the detector without affecting the patient’s line of sight in the visible range. High speed and automatic refractive power correction via the data form the Shack-Hartmann detector not only can eliminate the need for verbal feedback from the patient, but also can shorten the examination time while having good prescription accuracy. *In-vivo* operation of the phoropter is an important next step that should be investigated in order to completely characterize the system performance such as evaluation of optical reflection from retina (Supplementary note 5).

The setup can be further miniaturized using custom optics to fabricate an ultra-portable auto-phoropter with a binocular form factor. The low power requirement of the phoropter (less than 5 W) enables the use of batteries so that the entire setup can be controlled wirelessly by a cellphone. This makes the system more compact and more accessible in hard-to-reach areas. Deploying an infrared LED source can further reduce the IR exposure safety concerns, especially for young children. The HOEs can be redesigned to work with LEDs and accommodate the larger bandwidth of the LED. Using a calibrated data for each pump and deploying high speed and high-resolution servo motors can reduce the correction process to less than 5 seconds (Supplementary note 6).

In conclusion, we presented a hand-held RE measurement system with sphero-cylindrical correction power range of −10 to +10 diopters and with 0.1 diopter increments. The fluidic lenses developed in this study can be deployed in applications that need high speed ophthalmic correction such as virtual and augmented reality systems. This phoropter system can be furthered improved by adding tunable prism lenses to analyze binocular vision. Additionally, deploying more complex tunable lenses enables higher order aberration correction through the data provided form the Shack-Hartmann sensor. Finally, by incorporating auxiliary devices such as Maddox rods the system can become compact stand-alone personal care device for home monitoring, which could decrease the rate of visual impairment drastically.

## Methods

### Phoropter Setup

The setup schematic is depicted in Fig. 1a. The 785 nm laser source (Thorlabs LPS-785-FC) is collimated using Thorlabs F810FC-780 to create 7.5mm diameter beam. Laser beam is then folded toward the eye using 50:50 beam splitter (Thorlabs CCM1-BS013). The reflected IR light from retina is redirected by two HOEs into the Shack-Hartmann sensor (Thorlabs WFS300-14AR). The lens and pump containers are 3D printed using Formlabs Form 2 printer. The pumps are controlled by Hitec HS-5065MG digital servo motors. Cargille laser liquid is used as the fluid inside the lenses.

### Holographic Optical Elements (HOEs)

Holographic lenses used in this design were made based on volumetric holograms due to their capability of providing ~100% diffraction efficiency (DE). In order to achieve such high DE while having undistorted spherical beam from the HOE, same geometry should be used for HOE recording and readout. However, holographic materials (phase materials) are not sensitive to the wavelength of our design (*λ* = 785 nm). The only commercially available holographic material sensitive in IR region is photo-emulsion, which has low DE. To overcome this problem, IR sensitive silver halide photo-plates were used to record the understored HOE lens and then a contact copy was made onto Bayfol photopolymer (which provides >95% DE) using a 532 nm collimated laser beam.

### Preparation of PDMS membranes

Sylgard-184 silicone elastomer kit form Dow Corning was spin coated for 15 s at 500 r.p.m. on a two-inch flat glass substrate to create a 250 (±10) *μ*m thick uniform membrane. The substrate was then baked in the oven at 100 °C for 30 minutes. The thickness of the PDMS membranes are measured using a DEKTAK profilometer.

### Simulations

Optical simulations were performed using OpticSutio (Zemax LLC.) to characterize the lens optical powers and Zernike value. The surface curvature values were chosen based on the liquid volume changes in each lens and their aperture size. The mechanical deformation simulation to characterize the PDMS membranes and optimize their dimensions were performed using ANSYS Structures software.

## Electronic supplementary material

Supplementary Video 1

Supplementary notes
