# Supplementary material for: A new low-cost, compact, auto-phoropter for refractive assessment in developing countries
Source: Sci Rep. 2017 Oct 25;7:13990. doi: 10.1038/s41598-017-14507-5 (PMC5656604; doi:10.1038/s41598-017-14507-5)
Supplement: Supplementary file 2 — Supplementary notes [file 41598_2017_14507_MOESM2_ESM.pdf]

Supplementary notes

A new low-cost, compact, Auto-Phoropter for refractive assessment in developing countries

Babak Amirsolaimani[1,*], Gholam Peyman[1], Jim Schwiegerling[1], Arkady Bablumyan[2] and N. Peyghambarian[1,2]

[1]College of Optical Sciences, University of Arizona, Tucson, AZ 85721, USA

[2]TIPD, LLC, 1430 N. 6th Ave, Tucson, AZ 85705

*Corresponding Author: *babak@optics.arizona.edu*

Supplementary note 1: Calculating volume change

The volume change of spherical and cylindrical lenses is measured to calculate the relation between the surface curvature and fluid volume. These changes are used for simulations and comparison with the measured data. Equation S1 shows the spherical cap volume based on the radius of surface SAG *S* and aperture radius *a*. The volume of cylindrical segment is calculated using Equation S2 where *R* is radius of curvature, *H* is the lens length and *S* is the surface SAG.

$$V = \frac{\pi h}{6}(3a^2 + S^2) \qquad \text{(Equation S1)}$$

$$V = H[R^2 cos^{-1}\left(\frac{R-S}{R}\right) - (R-S)\sqrt{2RS - S^2}] \qquad \text{(Equation S2)}$$

Supplementary note 2: Calculating optometric constants

Optometric constants can be measured using Zernike values corresponding to focus *Z4*, oblique astigmatism *Z5*, and vertical astigmatism *Z6*[1]. The magnitude of defocus and astigmatism is calculated using Equations S3 and S4 respectively. Astigmatism angle can be calculated using Equation S5. As it can be seen from this equation, all astigmatic angles can be corrected using two cylindrical lenses placed at 45 degrees with respect to each other.

$$Defocus\ magnitude: \quad 2Z_4 \pm \sqrt{Z_5^2 + Z_6^2} \qquad \text{(Equation S3)}$$

$$Astigmatism\ magnitude: \quad \pm 2\sqrt{Z_5^2 + Z_6^2} \qquad \text{(Equation S4)}$$

$$Astigmatism\ angle: \quad \frac{1}{2} tan^{-1}(\frac{Z_5}{Z_6}) \qquad \text{(Equation S5)}$$

Supplementary note 3: Arizona eye model[2]

The Arizona eye model (Fig. S1) is used to calibrate the fluidic lenses and servo motor positions in the system. The eye model properties is presented in Table S1. The automatic correction process is optimized by placing different trial lenses in front of the eye model and measuring the required refractive correction using the phoropter in each case.

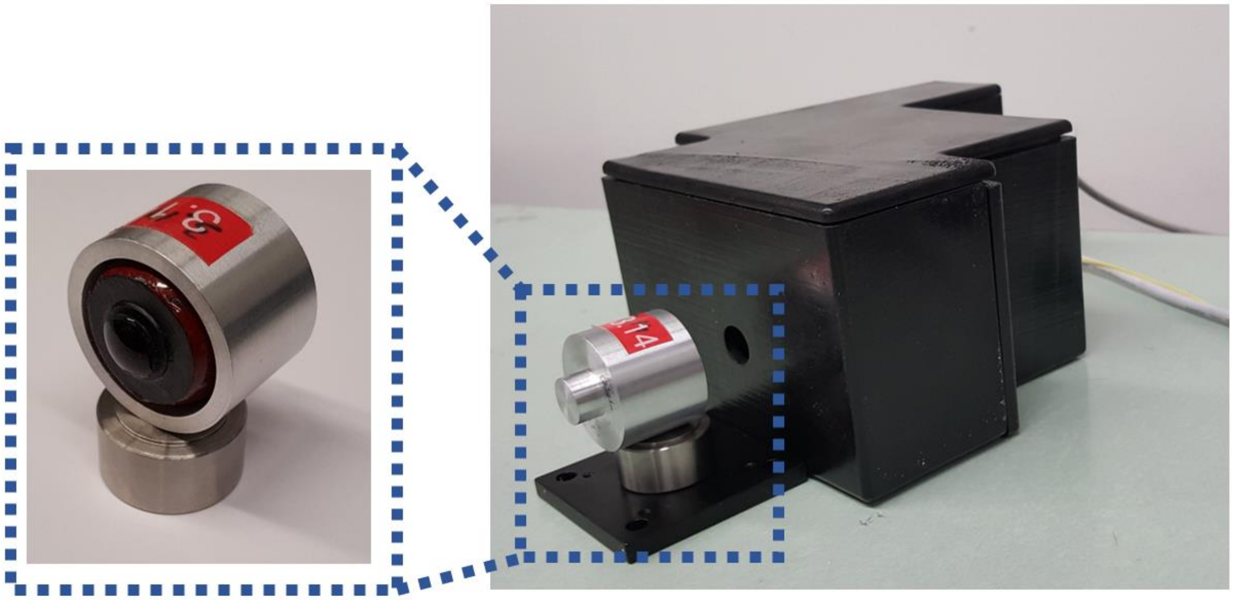

Figure S1. The Arizona eye model. Image of the eye model that used to calibrate the fluidic lenses and servo motor positions. The performance of the phoropter is investigated by placing various trial lenses in front of the eye model and optimizing the automatic correction process.

Table S1. Arizona eye model optical model properties

<table>
<tr><th>Layer</th><th>Radius (mm)</th><th>Conic (K)</th><th>Index (n)</th><th>Abbe (v)</th><th>Thickness (mm)</th></tr>
<tr><td></td><td rowspan="2">7.8</td><td rowspan="2">-0.25</td><td></td><td></td><td></td></tr>
<tr><td rowspan="2">Cornea</td><td rowspan="2">1.377</td><td rowspan="2">57.1</td><td rowspan="2">0.55</td></tr>
<tr><td rowspan="2">6.5</td><td rowspan="2">-0.25</td></tr>
<tr><td rowspan="2">Aqueous</td><td rowspan="2">1.337</td><td rowspan="2">61.3</td><td rowspan="2">2.97</td></tr>
<tr><td rowspan="2">12</td><td rowspan="2">-7.51</td></tr>
<tr><td rowspan="2">Lens</td><td rowspan="2">1.42</td><td rowspan="2">51.9</td><td rowspan="2">3.76</td></tr>
<tr><td rowspan="2">-5.2</td><td rowspan="2">-1.35</td></tr>
<tr><td rowspan="2">Vitreous</td><td rowspan="2">1.336</td><td rowspan="2">61.1</td><td rowspan="2">16.71</td></tr>
<tr><td rowspan="2">-13.4</td><td rowspan="2">0.00</td></tr>
<tr><td rowspan="2">Retina</td><td rowspan="2"></td><td rowspan="2"></td><td rowspan="2"></td></tr>
<tr><td></td><td></td></tr>
</table>

Supplementary note 4: Fluidic lens double passing

The effect of double passing through the fluidic lenses is compensated through the user interface software. Zemax simulations were done for both cases of single and double passing to evaluate the effects on each lens. Fig. S2a shows the dioptric power simulation of the spherical lens vs. surface SAG. The corresponding simulation results for cylindrical lens is depicted in Fig. S2b.

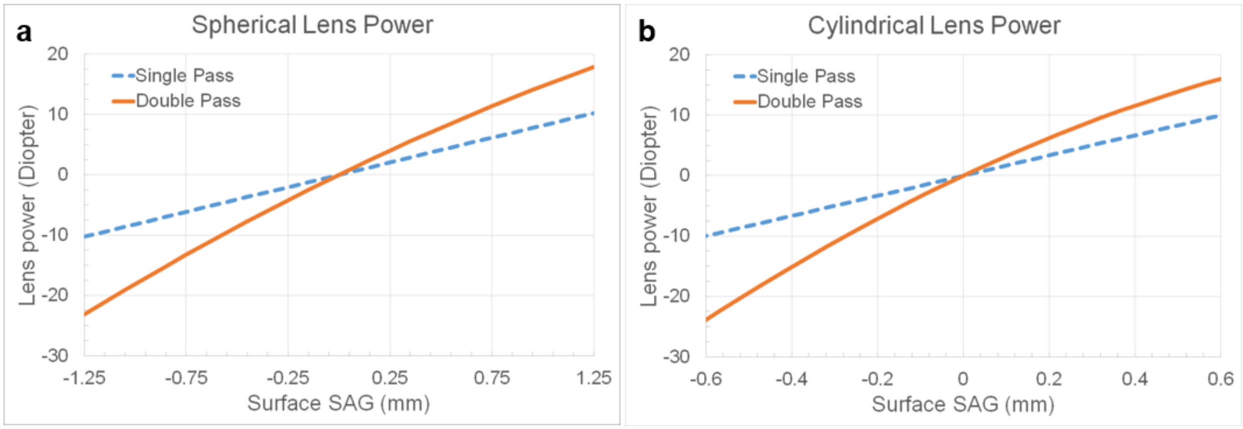

Figure S2. Single pass vs double pass. Zemax simulation results of the dioptric power comparison between single passing and double passing of the IR light through the **(a)** spherical lens and **(b)** cylindrical lens. The values in each case is considered in the user interface software to compensate the power difference.

Supplementary note 5: Optical transmission of the system

To characterize the optical transmission of the phoropter, power of the IR source was measured after each element. A pig eye was used to evaluated the amount of reflection form retina. Fig. S3a depicts the IR laser light path and the positions that optical power is measured. The power value of each position is illustrated in Fig. S3b. The reflection from pig eye retina is measure to be 0.2% to 0.5%.

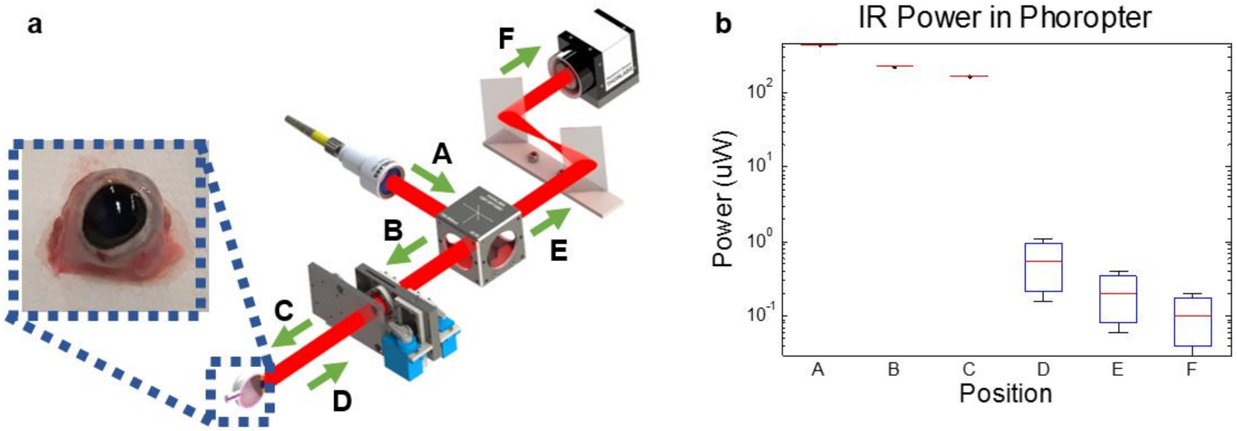

Figure S3. IR light power transmission inside the phoropter**. (a)** Schematic diagram of the setup and the positions that power was measured. A pig eye was used to evaluate the amount of reflection from retina. **(b)** The power value evaluated at each position inside the phoropter.

Supplementary note 6: Automatic correction calibration data

In order to speed up the automatic correction process, system can be calibrated based on the refractive error value at each servo motor position. Fig. S4 shows the relation of servo motor angular displacement to the optometric error for defocus and astigmatic lenses. The equation fitted on each curve is utilized to drive each servo motor in correction process to minimize the Zernike value and dioptric power.

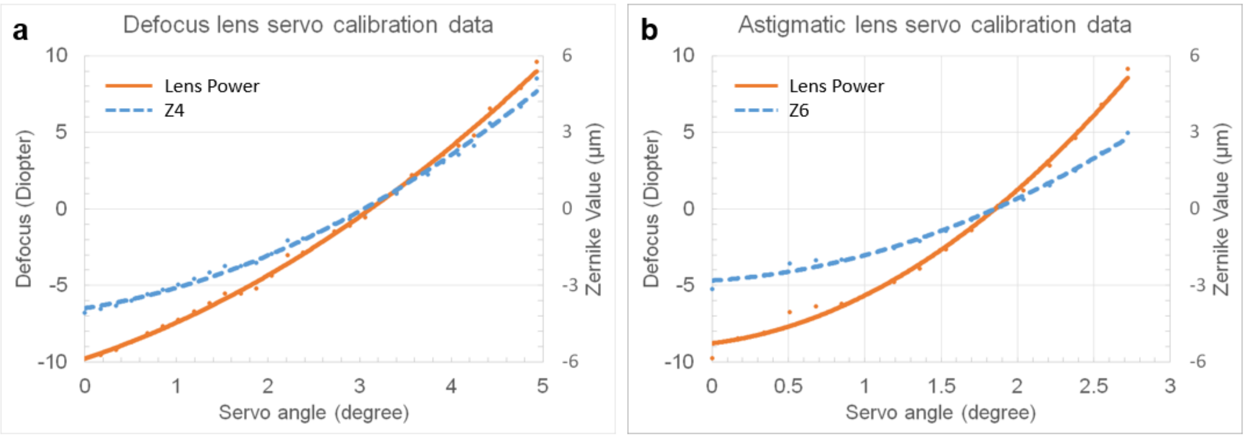

Figure S4. Lens calibration data vs servo motor position. **(a)** The spherical lens dioptric power and defocus Zernike value (Z4) at each servo motor position and **(b)** the corresponding data for the astigmatic lens.

References

1. James C. Wyant, and Katherine Creath, "Basic wavefront aberration theory for optical metrology," Applied optics and optical engineering 11.s 29 (1992).
2. Jim Schwiegerling, "Field Guide to Visual and Ophthalmic Optics," SPIE publications (2004).
